# Cervical cancer screening coverage and associated factors among women aged 25–59 in Eswatini: Insights from the 2021 Population-Based HIV Impact Assessment (PHIA)

**DOI:** 10.1371/journal.pone.0354508

**Published:** 2026-07-22

**Authors:** Abenezer Melaku Tafese, Bikiltu Dirbaba, Meseker Fentie, Charnita Zeigler-Johnson

**Affiliations:** 1 College of Population Health, Thomas Jefferson University, Philadelphia, Pennsylvania, United States of America; 2 Dornsife School of Public Health, Drexel University, Philadelphia, Pennsylvania, United States of America; 3 Fox Chase Cancer Center, Temple Health, Philadelphia, Pennsylvania, United States of America; University of California Davis School of Medicine, UNITED STATES OF AMERICA

## Abstract

**Background:**

Cervical cancer is the most common cancer and the leading cause of cancer mortality among women in Eswatini, which reported the highest cervical cancer incidence and mortality rates globally in 2022. However, recent nationally representative published data on cervical cancer screening coverage in Eswatini remain limited. This study aimed to estimate cervical cancer screening coverage and identify factors associated with screening uptake among women aged 25–59 years in Eswatini.

**Method:**

Data were drawn from the 2021 Eswatini Population-based HIV Impact Assessment (PHIA), a nationally representative cross-sectional household survey. Survey-weighted bivariable and multivariable logistic regression analyses were conducted to identify factors associated with screening uptake. Results are reported as adjusted odds ratios (AORs) with 95% confidence intervals (CIs).

**Result:**

Among 3,192 women aged 25–59 years, 48.2% reported ever having undergone cervical cancer screening. Higher odds of screening were observed among women living with HIV (AOR 2.26, 95% CI 1.93–2.65), those aged 36–45 years (AOR 1.35, 95% CI 1.12–1.62), and those in the fourth wealth quintile (AOR 1.40, 95% CI 1.05–1.88). Lower odds of screening were observed among women who were not married (AOR 0.65, 95% CI 0.54–0.78), with no formal education (AOR 0.56, 95% CI 0.39–0.80), no recent healthcare visit (AOR 0.67, 95% CI 0.55–0.83), no family planning use (AOR 0.82, 95% CI 0.69–0.97), and one sexual partners (AOR 0.78, 95% CI 0.65–0.94).

**Conclusion:**

Despite notable progress, cervical cancer screening coverage in Eswatini remains below World Health Organization targets. Enhancing community awareness, addressing stigma and structural barriers, and integrating cervical cancer screening into routine and reproductive healthcare services may accelerate progress toward achieving the WHO 2030 targets.

## Introduction

Cervical cancer remains a significant global public health concern, ranking as the fourth most common cancer and cause of cancer death among women, with an estimated 604,000 new cases and 342,000 deaths in 2022 [1]. The burden is disproportionately concentrated in low- and middle-income countries (LMICs), which account for approximately 94% of cervical cancer–related deaths, where the disease often remains the leading cause of cancer mortality among women [2,3]. Notably, 18 of the 20 countries with the highest cervical cancer burden globally are in Africa [4]. In sub-Saharan Africa, cervical cancer is the leading cause of cancer-related mortality and the second most frequently diagnosed cancer among women, accounting for nearly one-quarter of cancer incidence and mortality in the region [1].

Despite this substantial burden, progress in cervical cancer prevention and control across Africa remains limited. As of 2024, only 28 of the 47 WHO Member States in the African region have introduced HPV vaccination into their national immunization programs, and only five have achieved 90% coverage or higher [5]. In 2023, only 22% of girls turning 15 in Africa had received at least one dose of the HPV vaccine [6]. Cervical cancer screening coverage is similarly low, with only 14% of women aged 30–49 reporting ever having been screened [7].

Several sociodemographic, behavioral, and healthcare-related factors have been documented to influence cervical cancer screening uptake [8]. Exposure to mass media, higher educational attainment, and greater household wealth are consistently associated with higher screening participation [9,10]. Screening uptake has also been linked to cervical cancer awareness, perceived risk, and a history of sexually transmitted infections [8]. In contrast, rural residence, limited access to healthcare services, and the absence of healthcare provider recommendations are consistently associated with lower screening uptake [8,11].

Cervical cancer prevention in Africa is further challenged by the region’s high human immunodeficiency virus (HIV) prevalence, which accounts for approximately 65% of the global HIV burden [12]. HIV is a well-established risk factor for cervical cancer, and the proportion of cervical cancer cases attributable to HIV closely mirrors HIV prevalence. In high-prevalence settings, more than 40% of cervical cancer cases are linked to HIV, compared to less than 5% in areas with low HIV prevalence [13]. In sub-Saharan Africa, approximately one in four women with cervical cancer is living with HIV, and about 20% of cases are directly attributable to HIV infection [14].

Eswatini is a sub-Saharan African country with a disproportionately high burden of cervical cancer and HIV [1,15]. Cervical cancer is the most diagnosed cancer and the leading cause of cancer-related mortality among women in Eswatini, accounting for more than half of all cancer cases and deaths [1]. In 2022, the country recorded the highest age-standardized incidence and mortality rates of cervical cancer globally [1]. This elevated burden is strongly associated with the country’s high HIV prevalence, where one-quarter of adults aged 15–49 are living with HIV [15]. Cervical cancer also imposes a substantial economic burden in the country, with an estimated annual cost of US$19 million, largely attributable to direct medical expenditures for treating advanced-stage disease [16].

Eswatini has implemented several measures to strengthen cervical cancer prevention. The HPV vaccine was introduced into the national immunization program in June 2023 for girls aged 9–14 years, with coverage reaching 73% by August 2024, reflecting substantial progress in primary prevention [17]. However, progress in cervical cancer screening coverage remains limited. The 2018 national guideline recommends screening initiation at age 25 years or at the time of HIV diagnosis, with discontinuation at age 59 years [18]. Despite these recommendations, screening uptake has remained low, with only 19% of women aged 30–49 years having ever been screened in 2019 [19].

Up-to-date data are essential for tracking progress in cervical cancer prevention and informing public health strategies. However, recent data on cervical cancer screening coverage in Eswatini remain limited, hindering assessment of progress toward the World Health Organization’s 90–70–90 cervical cancer elimination targets by 2030 [20]. This study aimed to address this gap by estimating cervical cancer screening coverage among women aged 25–59 years and identifying factors associated with screening.

## Materials and methods

### Study design and data source

This study is a secondary analysis of the adult individual dataset from the 2021 Swaziland HIV Incidence Measurement Survey (SHIMS3), a nationally representative, cross-sectional household survey conducted as part of the Population-based HIV Impact Assessment (PHIA) Project. SHIMS3 aimed to assess HIV-related health indicators, including HIV prevalence, incidence, and service coverage, among individuals aged ≥15 years in Eswatini. Data was collected from May to November 2021 using a stratified, multistage probability sampling method. Stratification was based on urban versus rural residence within Eswatini’s four administrative regions, yielding eight sampling strata. The primary sampling units were enumeration areas (EAs) selected from the 2017 Eswatini Population and Housing Census. Households were randomly selected from each EA, and all eligible individuals aged 15 or older who had spent the previous night in the household were interviewed.

### Study population

The SHIMS3 2021 survey was conducted using a nationally representative sample of 7,000 households across Eswatini. Of these, 5,413 households (77.3%) were eligible and completed the household interview. Across these households, 25,132 individuals of all ages were rostered. Among them, 12,043 respondents aged 15 or older completed the individual interview, and 11,199 provided blood specimens. For the present study, we analyzed women aged 25–59 years (N = 3,192), the target population for cervical cancer screening under Eswatini’s 2018 guidelines [18].

### Variables

The primary outcome was self-reported ever undergoing cervical cancer screening, coded as a binary variable (Yes = 1, No = 0). Independent variables included age, administrative region, educational attainment, place of residence, marital status, household wealth quintile, employment (compensated work in the past 12 months), healthcare utilization in the past 12 months, current use of family planning, HIV status, and the number of lifetime sexual partners. HIV status was determined using the national testing algorithm, involving sequential rapid tests (Determine™ and Unigold™). All positive or inconclusive results were confirmed using the Geenius™ HIV 1/2 assay.

### Data analysis

Data analyses were conducted using SAS version 9.4. All analyses accounted for the complex survey design, including sampling weights, clustering, and stratification. Descriptive statistics were used to summarize participant characteristics. Associations between cervical cancer screening and independent variables were assessed using bivariable logistic regression. Variables with a p-value <0.20 in bivariable analyses, along with variables identified as potential confounders based on prior studies and contextual relevance, were considered for inclusion in the multivariable logistic regression model [21]. Multicollinearity was assessed using variance inflation factors (VIF), with VIF > 10 used as the cutoff for serious multicollinearity [22]. Model fit was evaluated using a survey-adjusted Archer–Lemeshow goodness-of-fit test. Adjusted odds ratios (AORs) with 95% confidence intervals (CIs) and two-sided p values were reported.

### Ethical considerations

This study used de-identified, publicly available data from SHIMS3. Ethical approval for the original survey was obtained from the Eswatini Ministry of Health and the institutional review boards of collaborating partners. Access to the data for this secondary analysis was granted by the International Center for AIDS Care and Treatment Programs (ICAP) at Columbia University. Because the dataset contained no personal identifiers, additional ethical approval was not required.

## Result

### Population characteristics

A total of 3,192 women aged 25–59 years were included in the analysis. Women aged 25–35 years constituted the largest age group (44.3%), followed by those aged 36–45 years (30.4%) and 46–59 years (25.3%). Over half of the participants were married (51.0%). The highest educational attainment for most women was secondary education (54.9%). More than half were unemployed (58.0%). The majority resided in rural areas (68.0%). Most women reported at least one healthcare visit in the past 12 months (78.1%) and current use of family planning services (58.4%). The majority reported having two or more lifetime sexual partners (74.6%). Nearly half were living with HIV (44.0%) (Table 1).

**Table 1:** Population characteristics and cervical cancer screening prevalence in Eswatini, 2021.

Variable	Category	Frequency Total N = 3192		Ever Screening Prevalence N = 1504 (48.2%)	
		Frequency	Weighted column Percent (%)	Frequency	Weighted Row Percent (%)
Age	25-35	1410	44.3	621	44.8
	36-45	917	30.4	511	56.5
	46-59	865	25.3	372	44.3
Marital status	Single	966	32.5	404	42.7
	Married	1710	51.0	841	50.7
	Divorced	249	8.9	135	55.1
	Widowed	267	7.6	124	47.3
Education	No school	217	6.2	81	39.5
	Primary	773	22.5	333	43.7
	Secondary	1741	54.9	859	50.2
	Higher	461	16.4	231	51.3
Employment	Yes	1215	42.0	601	50.8
	No	1977	58.0	903	46.3
Wealth quintile	Lowest	730	20.1	310	42.9
	Second	753	20.8	339	45.3
	Middle	688	21.3	332	48.9
	Fourth	556	19.8	283	53.1
	Highest	465	18.0	240	51.5
Residence	Rural	2538	68.0	1151	45.6
	Urban	665	32.0	354	53.7
Region	Hhohho	803	24.6	393	49.6
	Lubombo	643	18.5	310	49.8
	Manzini	1102	39.0	527	49.3
	Shiselweni	644	17.9	274	42.3
HC Utilization	Yes	2482	78.1	1260	51.8
	No	710	21.9	244	35.6
Family planning	Yes	1850	58.4	936	52.0
	No	1342	41.6	568	42.9
HIV status	Positive	1385	44.0	814	59.6
	Negative	1807	56.0	690	39.1
Sexual partner†	No partner	20	0.7	1	3.9
	One partner	858	24.7	345	40.7
	Two or more	2314	74.6	1158	51.1

***Notes*** : *Healthcare utilization in the past 12 months; †Number of lifetime sexual partners.

### Cervical cancer screening

Overall, 48.2% of women reported having undergone cervical cancer screening at least once in their lifetime. The prevalence of screening was higher among women living with HIV (59.6%) compared to HIV-negative women (39.1%) (Table 1).

### Factors associated with cervical cancer screening uptake

Women aged 36–45 years had higher odds of cervical cancer screening compared with women aged 25–35 years (AOR = 1.35, 95% CI: 1.12–1.62). Single women had lower odds of screening than married women (AOR = 0.65, 95% CI: 0.54–0.78). Women with no formal education and those with primary education had lower odds of screening than women with secondary education (AOR = 0.56, 95% CI: 0.39–0.80; AOR = 0.76, 95% CI: 0.61–0.95, respectively). Screening was more likely among women in the fourth wealth quintile compared with those in the lowest quintile (AOR = 1.40, 95% CI: 1.05–1.88). Although employment, place of residence, and region were associated with screening in bivariable analyses, these associations were not retained after multivariable adjustment (Table 2).

**Table 2:** Factors associated with cervical cancer screening in Eswatini, 2021.

Variable	Category	COR (95%CI)	p-value	AOR (95%CI)	p-value
Age	25-35	1(Ref)		1(Ref)	
	36-45	1.60(1.34–1.90)	0.000	1.35 (1.12–1.62)	0.002
	46-59	0.98(0.81–1.19)	0.827	1.13 (0.90–1.41)	0.299
Marital status	Not married	0.73(0.61–0.86)	0.000	0.65 (0.54–0.78)	0.000
	Married	1(Ref)		1(Ref)	
	Divorced	1.19(0.95–1.49)	0.121	0.92 (0.74–1.13)	0.415
	Widowed	0.88(0.65–1.17)	0.370	0.80(0.56–1.15)	0.224
Education	No school	0.65(0.47–0.90)	0.010	0.56 (0.39–0.80)	0.002
	Primary	0.77(0.64–0.93)	0.008	0.76 (0.61–0.95)	0.016
	Secondary	1(Ref)		1(Ref)	
	Higher	1.04(0.81–1.35)	0.740	1.18 (0.90–1.54)	0.230
Employment	Yes	1(Ref)		1(Ref)	
	No	0.83 (0.72–0.97)	0.020	0.93(0.78–1.10)	0.392
Wealth quintile	Lowest	1(Ref)		1(Ref)	
	Second	1.10(0.88–1.39)	0.397	1.15 (0.91–1.46)	0.246
	Middle	1.28(0.98–1.66)	0.070	1.16 (0.88–1.53)	0.294
	Fourth	1.51(1.16–1.95)	0.002	1.40 (1.05–1.88)	0.024
	Highest	1.41(1.05–1.91)	0.024	1.30 (0.91–1.84)	0.144
Residence	Rural	1(Ref)		1(Ref)	
	Urban	1.38(1.14–1.68)	0.002	1.19 (0.97–1.45)	0.124
Region	Hhohho	1.00(0.82–1.25)	0.933	1.18 (0.94–1.49)	0.148
	Lubombo	1.02(0.80–1.30)	0.870	1.24 (0.96–1.60)	0.092
	Manzini	1(Ref)			
	Shiselweni	0.75(0.59–0.96)	0.021	0.87 (0.67–1.12)	0.275
HC utilization*	Yes	1(Ref)		1(Ref)	
	No	0.52(0.42–0.63)	0.000	0.67 (0.55–0.83)	0.000
Family planning	Yes	1(Ref)		1(Ref)	
	No	0.70(0.60–0.81)	0.000	0.82 (0.69–0.97)	0.025
HIV status	Positive	2.32(2.04–2.65)	0.000	2.26 (1.93–2.65)	0.000
	Negative	1(Ref)		1(Ref)	
Sexual partner^†^	No partner	0.04(0.01–0.33)	0.003	0.07 (0.01–0.63)	0.018
	One partner	0.66(0.56–0.78)	0.000	0.78 (0.65–0.94)	0.010
	Two or more	1(Ref)		1(Ref)	

Notes: COR = Crude Odds Ratio; AOR = Adjusted Odds Ratio; Ref = Reference category.*Healthcare utilization in the past 12 months; †Number of lifetime sexual partners.

Women without a healthcare visit in the past 12 months and those not using family planning services had lower odds of screening compared with women who had at least one healthcare visit and those using family planning services, respectively (AOR = 0.67, 95% CI: 0.55–0.83; AOR = 0.82, 95% CI: 0.69–0.97). Women with one partner or no partner had lower odds of screening than those with two or more partners (AOR = 0.78, 95% CI: 0.65–0.94; AOR = 0.07, 95% CI: 0.01–0.63, respectively). Women living with HIV had higher odds of screening than HIV-negative women (AOR = 2.26, 95% CI: 1.93–2.65) (Table 2).

No evidence of serious multicollinearity was observed, with all VIF values below 2. The survey-adjusted Archer–Lemeshow goodness-of-fit test indicated adequate model fit.

## Discussion

Our study found that cervical cancer screening coverage among women aged 25–59 years in Eswatini was 48.2%. This estimate is comparable to a prior study conducted in four primary healthcare clinics in Eswatini, which reported cervical cancer screening uptake of 44% [18]. Although our estimate represents an improvement compared with the 19% screening coverage reported in 2019, it remains below the World Health Organization target of 70% screening with a high-performance test [19,20]. This gap is particularly important because cervical cancer screening in Eswatini still commonly relies on visual inspection methods, while screening with high-performance tests remains below 5% [18,20,23]. Therefore, the observed coverage should be interpreted cautiously, as it may overestimate progress toward the WHO elimination targets.

Women living with HIV had higher cervical cancer screening uptake than HIV-negative women. This association may reflect the priority given to women living with HIV and the integration of screening into HIV care [13,24]. Such prioritization is warranted, given their six-fold higher risk of developing cervical cancer and greater likelihood of developing the disease at a younger age [13]. However, the close linkage of cervical screening with HIV service may inadvertently foster stigma and discourage screening among women without HIV. A prior study in Eswatini found that some women sought screening outside their communities rather than at local health facilities to protect their privacy and avoid judgment or assumptions about their HIV status [25]. Therefore, while prioritizing women living with HIV remains essential, expanding cervical cancer screening to the broader population through routine primary care and reproductive health encounters, while addressing privacy- and stigma-related barriers, may help increase participation among women without HIV.

Age was also associated with cervical cancer screening uptake. Women aged 36–45 years reported higher screening uptake than those aged 25–35 years, while uptake appeared slightly lower among women aged 46–59 years. This finding is consistent with studies from Ethiopia [26], Kenya [27], and pooled analysis across sub-Saharan African countries [28,29], which have identified age as an important determinant of cervical cancer screening uptake. The higher screening rate among women aged 36–45 years may reflect greater engagement with women’s health services, increasing opportunities for provider recommendation and referral. In contrast, the slightly lower uptake among women aged 46–59 years may reflect lower perceived risk, competing health priorities, and reduced contact with reproductive health services [18,30].

Disparities in cervical cancer screening were also evident by educational level and economic status. Women with no formal education had significantly lower odds of screening than those with secondary or higher education, consistent with studies from Eswatini [31] and other settings reporting higher screening uptake among women with greater educational attainment [28,32,33]. This association may reflect the role of education in improving health literacy, awareness of cervical cancer risk, and understanding of health information from providers and media sources, thereby facilitating screening uptake [28,29]. Women from wealthier households also had higher odds of screening, consistent with prior studies showing a positive association between household wealth and screening utilization [27,34,35]. One study found that some women avoid screening because they fear a positive diagnosis and were concerned about the affordability and accessibility of treatment; for many, fear of cervical cancer exceeded fear of HIV, partly because HIV treatment was perceived as more accessible [36]. This may be partly attributable to the absence of in-country radiotherapy services for invasive cervical cancer, which necessitates cross-border referral and creates substantial financial and logistical barriers to care [4,37].

Marital status and sexual history were also associated with cervical cancer screening uptake. Single women had lower odds of screening than married women, consistent with findings from Eswatini and other similar settings showing lower screening uptake among unmarried women [18,32,38]. Women with only one lifetime sexual partner also had lower odds of screening than those reporting two or more partners, consistent with regional evidence linking multiple sexual partners with higher screening uptake [39]. These patterns may reflect differences in perceived risk and contact with reproductive health services. Married women and women with multiple sexual partners may have more frequent interactions with reproductive or sexual health services, including contraception, STI testing, or counseling, which can create opportunities for screening referral or recommendation [18,29,32,38–40]. In contrast, unmarried women and those with fewer lifetime sexual partners, particularly younger women, may perceive themselves to be at lower risk and may therefore delay or forgo screening [18,29,32,38–40].

Recent healthcare utilization and family planning use also emerged as important predictors of cervical cancer screening, indicating the importance of regular contact with the healthcare system for preventive care. Routine engagement with health services may increase screening uptake by creating opportunities for provider recommendation, counseling, and referral. In Eswatini, provider recommendation is particularly important, as lack of advice from a doctor or nurse has been identified as a common reason for non-screening [18]. Family planning and other reproductive health services also offer important opportunities to expand cervical cancer screening and reach women who may otherwise have limited contact with the healthcare system [41,42]. Studies indicate that limited healthcare contact remains a major barrier to cervical cancer screening, whereas regular engagement, particularly through reproductive health services, enhances opportunities for screening and preventive counseling [29,32,43].

Taken together, our findings shows that cervical cancer screening uptake in Eswatini is shaped by the interplay of sociodemographic factors, cancer risk perception, and women’s access to the healthcare system. Lower educational attainment and limited economic resources may reduce awareness of cervical cancer screening and make it more difficult for women to overcome financial and logistical barriers to follow-up and treatment. Unmarried younger women and women with one sexual partner may perceive themselves to be at lower risk and may have fewer healthcare encounters that provide opportunities for screening recommendations.

To address the existing gaps, Eswatini is implementing a costed national plan to reach the WHO 90–70–90 cervical cancer elimination targets by 2030. The Eswatini National Cervical Cancer Elimination Acceleration Plan 2024–2030 provides a roadmap for expanding HPV vaccination, transitioning toward HPV DNA-based screening, integrating screening with HIV and women’s health services, and strengthening treatment capacity [23]. Ongoing community awareness and outreach activities are also key strategies to improve screening uptake, early detection, and timely treatment. However, translating these plans into measurable progress will require strong leadership, adequate financing, and robust monitoring and evaluation.

### Strengths and limitations

To our knowledge, this study presents the most recent population-based estimates of cervical cancer screening coverage in Eswatini and provides a comprehensive assessment of sociodemographic, behavioral, and clinical factors associated with screening uptake. However, some limitations should be acknowledged. Cervical screening status was self-reported, which may have introduced recall or social desirability bias. Information on the type of screening test used was not collected, limiting our ability to assess screening quality or the use of high-performance methods. The dataset also lacked information on other potentially relevant factors, including cervical cancer knowledge, perceived risk, partner support, history of sexually transmitted infections, and contextual factors that may influence screening behavior. Lastly, given the cross-sectional study design, the observed associations should be interpreted cautiously and cannot establish causal relationships.

## Conclusion

This study provides up-to-date data on cervical cancer screening coverage and its determinants among women in Eswatini, a country with a high burden of both cervical cancer and HIV. While improvements in screening uptake are encouraging, coverage remains below WHO targets with persistent socioeconomic disparities. Given the high HIV burden in Eswatini, continued prioritization of women living with HIV is important, alongside efforts to expand equitable access to screening for all women. Integrating cervical screening into women’s health services and addressing stigma, privacy issues, and structural barriers are critical steps toward improving screening coverage. Further research is needed to better understand women’s awareness and attitudes toward cervical cancer screening and access to high-performance screening tests in Eswatini.
